# The spatial and environmental distribution of the global eddy covariance tower network

**DOI:** 10.1007/s00484-026-03183-8

**Published:** 2026-03-30

**Authors:** Paul C. Stoy, Housen Chu, Emma Dahl, Daniela S. Cala, Victoria Shveytser, Susanne Wiesner, Ankur R. Desai, Kimberly A. Novick

**Affiliations:** 1 Department of Biological Systems Engineering, University of Wisconsin – Madison, Madison, WI USA; 2 Climate and Ecosystem Sciences Division, Lawrence Berkeley National Laboratory, Berkeley, CA USA; 3 O’Neill School of Public and Environmental Affairs, Indiana University, Bloomington, IN USA; 4 Department of Forest and Wildlife Ecology, University of Wisconsin – Madison, Madison, WI USA; 5 Department of Environmental and Resource Engineering, Technical University, Kongens Lyngby, Denmark; 6 Department of Atmospheric and Oceanic Sciences, University of Wisconsin– Madison, Madison, WI USA

**Keywords:** Carbon dioxide, Climate, Eddy covariance, Soil chemistry, Soil texture

## Abstract

Eddy covariance has revolutionized our understanding of ecosystem-atmosphere interactions. Multiple studies have characterized the climate space occupied by flux tower networks, but none to our knowledge have characterized if eddy covariance sites represent the global distribution of soil characteristics that are critical for determining ecosystem function or studied the distances between towers to apply ‘paired’ tower studies. Of 1233 global eddy covariance towers explored here, half had a nearest neighbor within 10 km. Soil database pixels with towers have nearly 20% more silt and 8% less sand than the global soil texture distribution, with more soil N (0.58 g/kg vs. 0.38 g/kg) and organic C (8.3 g/kg vs. 5.4 g/kg), and 10% greater cation exchange capacity in upper layers than pixels without towers. Global syntheses of eddy covariance towers should be cognizant that tower networks capture more fertile soils than the terrestrial surface on average. A logical way to improve global representativeness is to further build collaborations and invest in underrepresented regions.

**Supplementary Information:**

The online version contains supplementary material available at 10.1007/s00484-026-03183-8.

## Introduction

The eddy covariance technique has been indispensable for understanding the role that ecosystems play in the earth system by controlling the fluxes of water, carbon dioxide and other greenhouse gasses, ozone and other atmospherically active gases, heat, and momentum with the atmosphere (Baldocchi 2008; Clifton et al. 2020). As eddy covariance networks have grown, we have had more opportunities to understand how different ecosystems impact these fluxes to help us quantify the critical services that they provide (Novick et al. 2022).

Eddy covariance networks represent a combination of ‘coalitions of the willing’ with individual PIs or groups that share data (Novick et al. 2018), alongside organized tower networks designed with ecological and/or geographical representativeness in mind (Hargrove et al. 2003; Schimel et al. 2007; Beringer et al. 2016; Rebmann et al. 2018; Cleverly et al. 2019; Heiskanen et al. 2022). Eddy covariance towers tend to be clustered in regions with more research infrastructure (Chu et al. 2017; Pastorello et al. 2020) including regional tower clusters designed with specific research questions in mind (Butterworth et al. 2021; Runkle et al. 2017). It is well known that the global eddy covariance network does not capture the full range of observed global climates and ecosystem types (Stoy et al. 2009; Xiao et al. 2012), with consequences for ongoing efforts to extend the spatial and temporal representativeness of flux observations to larger scales, known as ‘upscaling’ (Jung et al. 2009, 2020).

In these efforts, less attention has been paid to edaphic variability (Pallandt et al. 2022) despite the central role played by soils in carbon, water, and nutrient cycling (Janzen 2004; Chapin et al. III 2009). Few if any studies have characterized mean distances between towers to understand how they may be clustered geographically for a ‘paired’ tower design (Baldocchi 2014). We seek to address these discrepancies by characterizing the physical environment of the global eddy covariance tower network across climate, space, and ecosystem type, with a focus on the edaphic characteristics that often go understudied in analyses of surface-atmosphere exchange, to contribute to the common goal of continuously improving flux networks (Papale 2020; Xiao et al. 2025).

## Materials and methods

### Eddy covariance tower locations

We queried the Ameriflux, CarboEurope, AsiaFlux, TERN, OzFlux, CarboAfrica, LaThuile, and FLUXNET2015 databases, in many cases with assistance from local experts (see Acknowledgements) to create a list of locations where eddy covariance measurements have been made (Supporting Information). We subsequently call these ‘towers’. We exclude inland and open water for which databases do not provide estimates of soil characteristics and note that many towers are privately or Tribally owned with locations that are not in the public domain such that our analysis is an underrepresentation of all locations that have or have had eddy covariance towers.

### Global climate and plant functional type

We seek to quantify the representativeness of eddy covariance tower placement with respect to continent, global climatic and edaphic conditions, and International Geosphere Biosphere Program plant functional type (PFT). Mean annual temperature (MAT) and precipitation (MAP) data were available from the metadata of many tower sites, but not others. To approximate climatic conditions at these sites, and to add mean annual incident solar radiation (SRAD) to our analysis, we used observations from the WorldClim 2.1 database of climate normals for the period 1970–2000 (Fick and Hijmans 2017). Monthly climate data within the ~ 1 km resolution Worldclim 2.1 pixels that included towers were compiled using R (R Core Team 2022) (Table S1) and aggregated to annual averages. Elevation above sea level data were available for many towers, but if not was estimated using data from the *elevatr* package in R (Hollister et al. 2022) that accesses the Amazon Web Service Terrain Tiles, Open Topography Global Datasets, and USGS Elevation Point Query Service. PFT was collected from tower sites when available and estimated using a global distribution of PFTs compiled by Loveland et al. (2000) when not, and we report the fraction of global PFTs from Loveland et al. (2000) for comparison. We used a random draw of 10,000 data points on the sphere using the ‘runifsphere’ command in the *globe* package in R (Baddeley et al. 2017) to estimate the global distribution of terrestrial climatic conditions from WorldClim2.1 and elevation from *elevatr* to compare tower information with a representative global distribution of climate and geographical attributes from the terrestrial surface. We use tower-reported values when possible and observations from global databases when necessary and provide information on each in Table S1. Uncertainties in applying global database information instead of tower observations (Vuichard and Papale 2015) are discussed below.

### Soil characteristics

One may argue that edaphic conditions are used too infrequently to interpret eddy covariance observations despite the central role of soils in ecosystem functioning. We approximated edaphic conditions at each site using the 250 m SoilGrids 2.0 database (Poggio et al. 2021) assisted by the *soilDB* package (Beaudette et al. 2022) in R. We used data from the uppermost soil layer (0–5 cm) for simplicity and explored the mean estimated value of sand, silt, and clay fraction (in percent), pH, N concentration, soil organic matter content (in the fine soil fraction), bulk density, and cation exchange capacity. Soil and sedimentary deposit thickness for pixels that include towers were taken from the database of Pelletier et al. (2016) (Table S2). We note that our results are subject to the uncertainty of global soil databases and their interpolation routines. Observations from tower locations are compared against a global distribution of all quantities approximated by a random sample on the sphere (Baddeley et al. 2017) from 10,000 locations from SoilGrids 2.0 and the Pelletier et al. (2016) database. Distributions of global and tower sand, silt, and clay fractions were plotted in the soil texture triangle using the *soiltexture* package in R (Moeys 2018). Figures are otherwise plotted using *matplotlib* (Hunter 2007) in Python.

### Uncertainty and statistical analysis

There are multiple sources of uncertainty when attempting to quantify where towers exist across geographic, climate, and edaphic space. These include errors associated with compiling the ever-changing suite of tower locations (e.g. Table S1), errors in soil and climate estimates from global databases including subpixel variability, uncertainty due to the extrapolation approach of the global databases (Fick and Hijmans 2017; Poggio et al. 2021), and uncertainties in tower measurements themselves including climatic and edaphic variability within the flux footprint and the representativeness of the flux footprint within the pixel dimensions (Chu et al. 2021). To help quantify the degree of uncertainty induced by the latter factors, we compare climate data reported from tower sites against that from WorldClim 2.1, noting that climate normals themselves of course are changing due to global climate change (Rigal et al. 2019). Meteorological data products tend to be sufficiently related to eddy covariance measurements to be used for gapfilling missing tower meteorological data (De Canniére et al. 2026) but discrepancies may exist, especially in areas characterized by rapid climate changes over short distances including mountainous regions. No eddy covariance towers have been operational during the full period used to calculate the 30-year climate normals from WorldClim 2.1, so differences between tower and climate database records should be expected.

To demonstrate the range of soil texture values that may be encountered when using different soil datasets, and to help validate soil characteristic results for a subset of sites, we also study soil silt, sand, and clay content from the CHEESEHEAD19 database of 19 eddy covariance towers within a 10 × 10 km domain in northern Wisconsin, USA (Butterworth et al. 2021) using the average of three measurements from soil cores within the flux footprints (Hu et al. 2020), data from the NRCS soil survey (Shveytser et al. 2024), and data from SoilGrids 2.0 (Poggio et al. 2021).

The Bonferroni correction was applied when interpreting statistical differences between global and tower climate and edaphic characteristics using Welch’s two-sided t-tests at the 𝛼 < 0.05 level. Strictly speaking, tower locations are a subset of the global datasets, but with a global terrestrial area of 148,326,000 km^2^ and data drawn from 1233 tower point locations in pixels that are 1 km^2^ or smaller, the chance of resampling is less than 1 in 100,000. We therefore assume that the tower and global data distributions are statistically distinct.

## Results

### Tower location and plant functional type

**Fig. 1:**
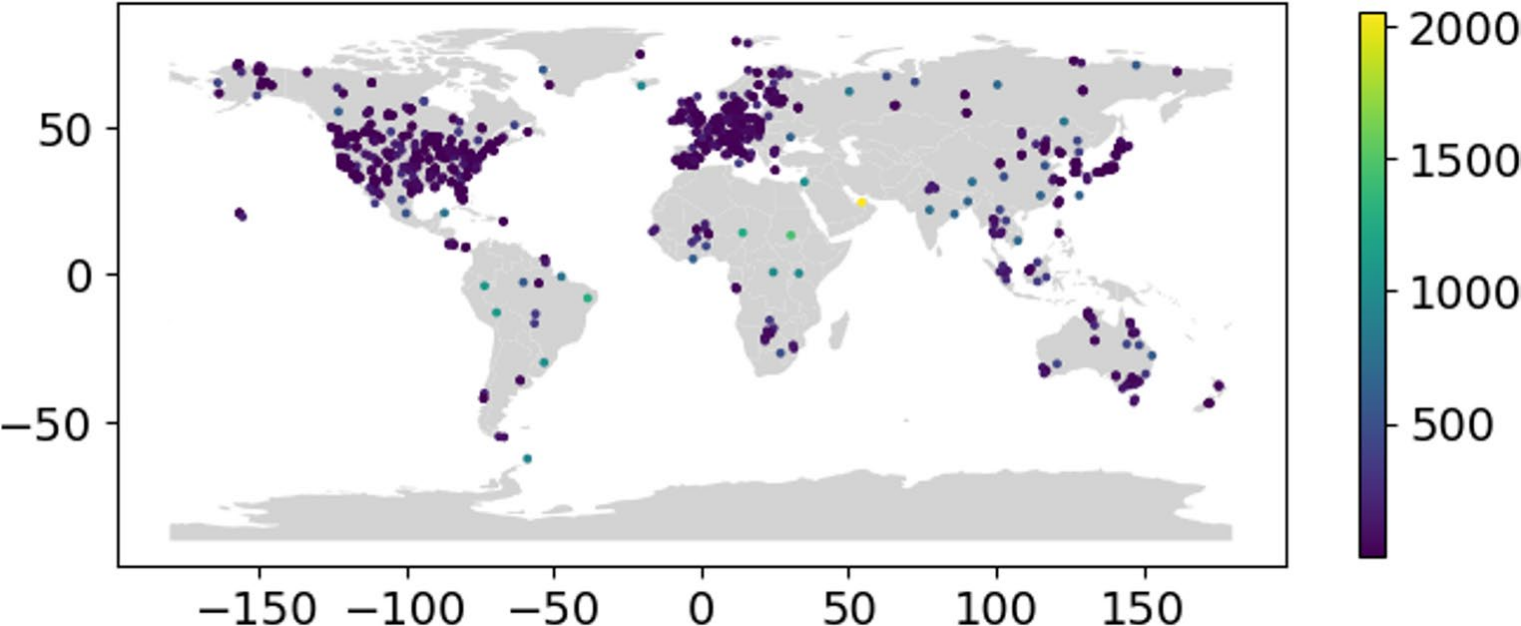
A map of the location of eddy covariance towers in our combined database (Table S1) colored with respect to distance to nearest neighboring tower in km

At the present, towers that have been included in global databases are absent from large expanses of the terrestrial biosphere including much of Africa, the Middle East, Central and South Asia, South America, and Siberia (Fig. 1). There are relatively few towers in savannas and woody savannas, open shrublands, evergreen broadleaf forests, snow & ice - dominated ecosystems, and barren-sparse vegetation ecosystems than the global distribution of these IGBP categories (Table 1). Of continents with the most eddy covariance towers, North America has more towers (575) than Europe (395 and 192, 48.6%) but a lower density of total towers (2.37 × 10^− 5^ km^− 2^ vs. 3.95 × 10^− 5^ km^− 2^) (Table 2). Of the 1233 towers that we compiled from global databases (Table S1), 626 (51%) had a nearest neighbor within 10 km and 1063 (86%) had a nearest neighbor within 100 km (Figure S1).

**Table 1:** The percent of the terrestrial surface and eddy covariance tower locations with different IGBP vegetation types from Loveland et al. (2000), and their percent difference. The total number of eddy covariance towers is listed in parentheses. Inland water bodies do not cleanly map onto IGBP vegetation types and comprise some 359 million ha globally (Bastin et al. 2019). Note that we did not sample towers measuring inland water for this analysis as we felt the soils databases would not represent these accurately

IGBP vegetation type	IGBP Abbreviation	Globe	Towers	Difference (Towers - Globe)
Evergreen needleleaf forest	ENF	4.3%	14.4% (178)	10.1%
Evergreen broadleaf forest	EBF	8.3%	4.9% (60)	-3.4%
Deciduous needleleaf forest	DNF	1.3%	1.5% (19)	0.2%
Deciduous broadleaf forest	DBF	2.2%	9.3% (115)	7.1%
Mixed forest	MF	4.3%	3.1% (38)	-1.2%
Closed shrubland	CSH	1.8%	1.5% (18)	-0.3%
Open Shrubland	OSH	12.4%	4.5% (55)	-7.9%
Woody Savanna	WSA	7.0%	1.1% (14)	-5.9%
Savanna	SAV	6.4%	2.6% (32)	-3.8%
Grassland	GRA	7.6%	16.5% (203)	8.9%
Wetland	WET	0.9%	13.7% (169)	12.8%
Crop	CRO	19.2%	20.4% (251)	1.2%
Urban	URB	0.2%	2.8% (34)	2.6%
Snow/Ice	SNO	11.3%	0.2% (3)	-11.1%
Barran	BSV	12.7%	1.4% (17)	-11.3%
Water	Water	-	1.2% (15)	-

**Table 2:** The number (density) of eddy covariance towers included in our global database (Table S1) by continent

Africa	32 (1.05 × 10^− 6^ km^− 2^)
Antarctica	1 (7.04 × 10^− 8^ km^− 2^)
Asia	160 (3.59 × 10^− 6^ km^− 2^)
Europe	396 (3.95 × 10^− 5^ km^− 2^)
Oceania	49 (5.76 × 10^− 6^ km^− 2^)
North America	575 (2.37 × 10^− 5^ km^− 2^)
South America	20 (1.12 × 10^− 6^ km^− 2^)

### Climate

**Fig. 2:**
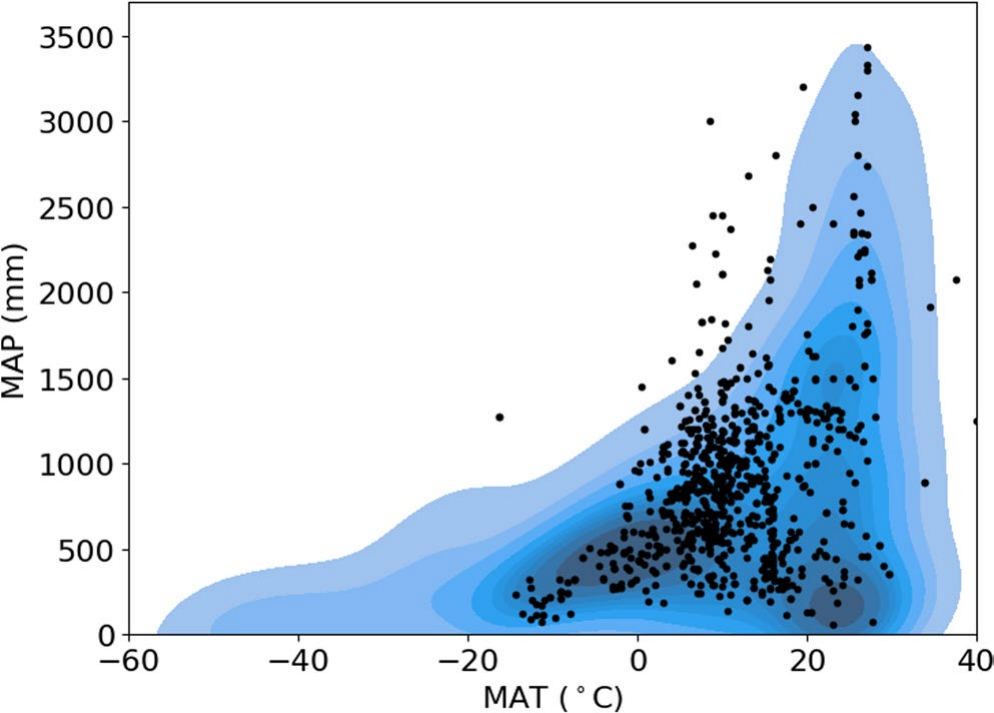
The mean annual temperature and precipitation of eddy covariance tower sites versus a two-dimensional kernel density estimate of global temperature and precipitation from the WorldClim 2.1 database (Fick and Hijmans 2017)

**Fig. 3:**
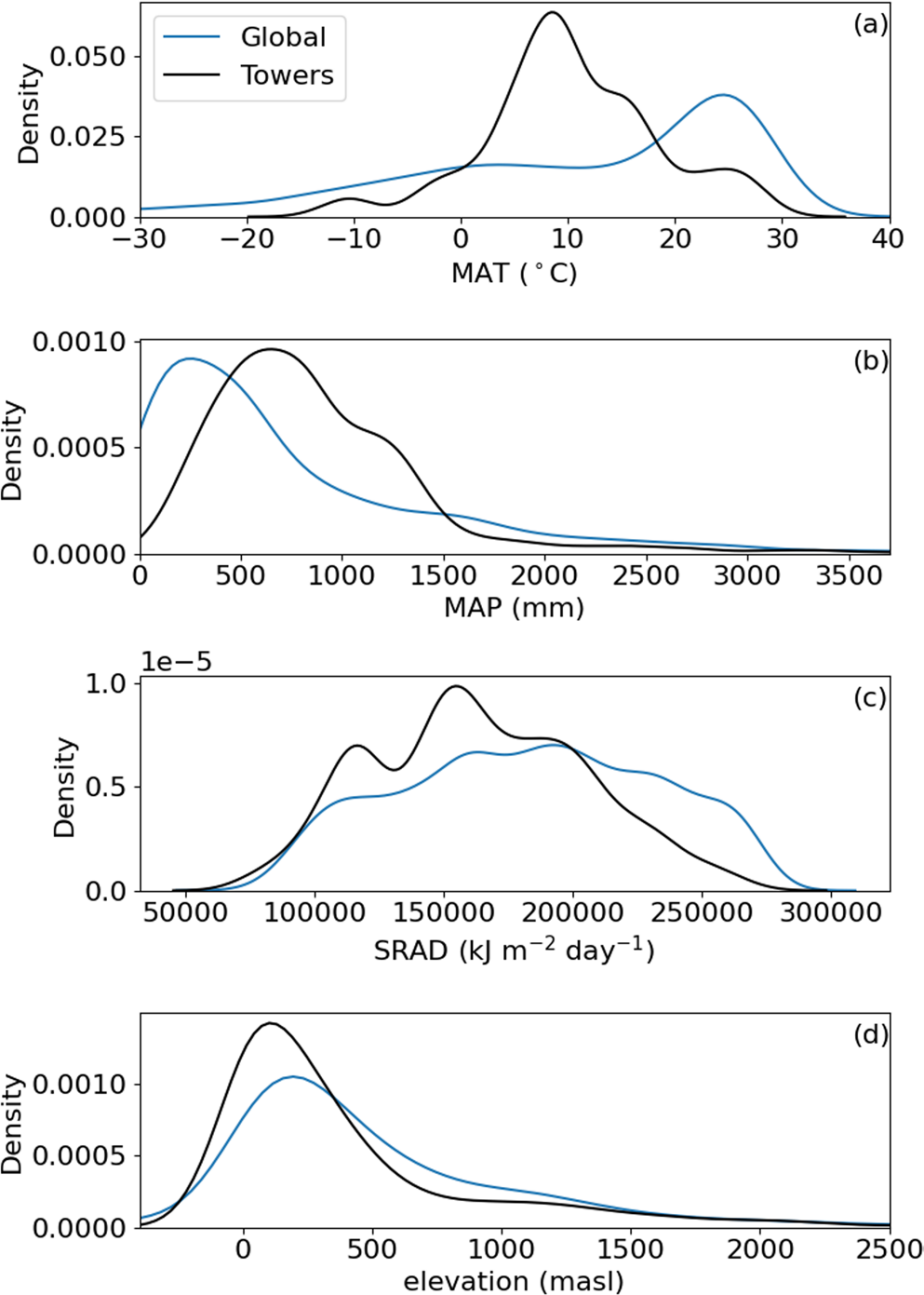
Probability density functions of mean annual temperature (MAT, **a**), mean annual precipitation (MAP, **b**) and the average daily sum of incident solar radiation (SRAD, **c**) for a random sample of 10,000 points from WorldClim 2.1 (Fick and Hijmans 2017) and elevation (**d**) from *elevatr* (Hollister et al. 2022) for the global terrestrial surface (‘Global’) and eddy covariance tower locations (‘Towers’, Table S1)

**Fig. 4:**
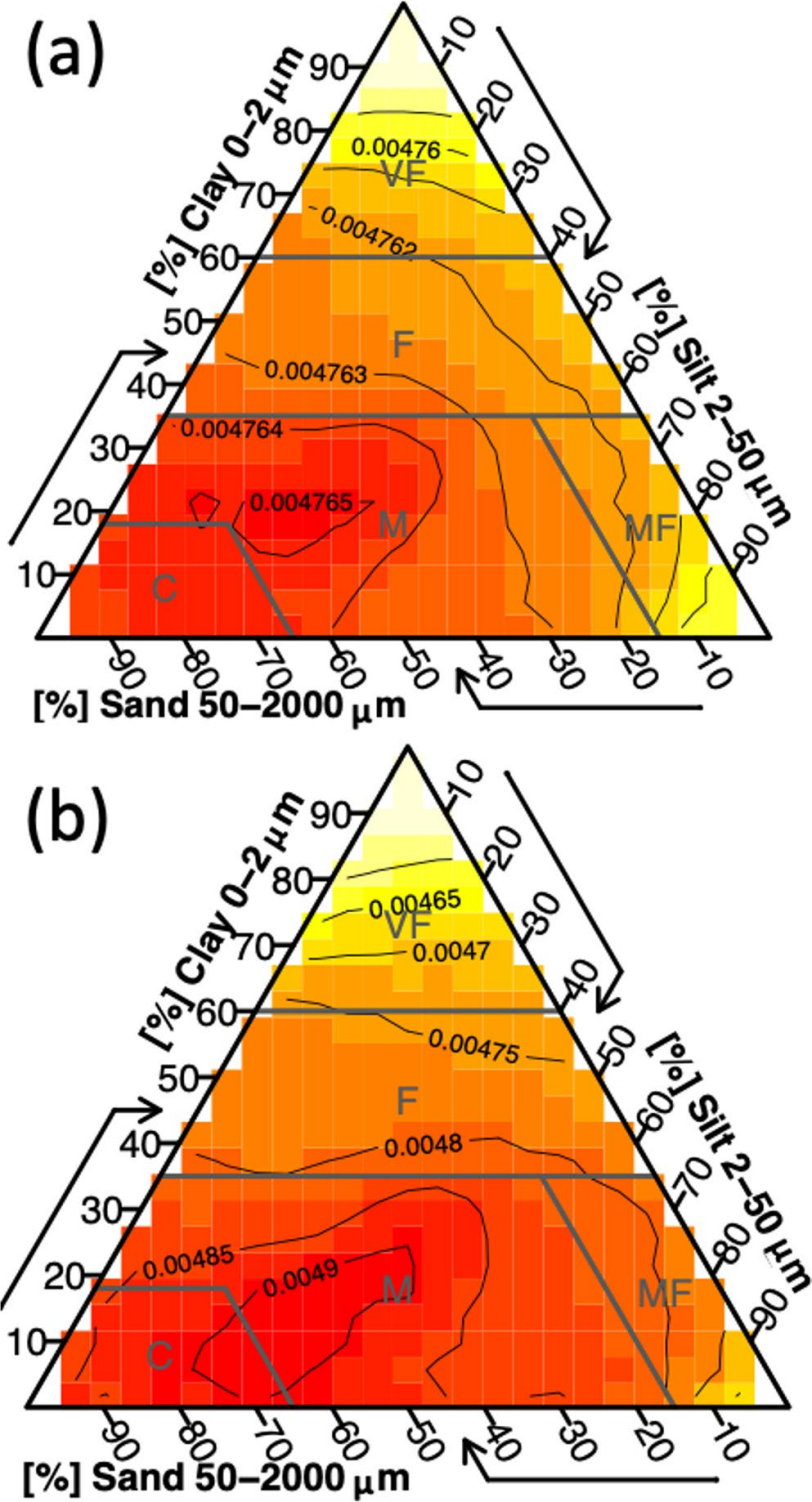
A heat map of the global distribution of soil texture generated by drawing (**a**) 10,000 random samples, and (**a**) data from eddy covariance towers from the SoilGrids database (Poggio et al. 2021). Figures were generated using the *soiltexture* package (Moeys 2018) in R

Towers in very cold, hot, dry, and wet ecosystems, tend to be underrepresented in global databases (Figs. 2 and 3), which is dominated by temperate ecosystems (Stoy et al. 2009; Xiao et al. 2012; Pastorello et al. 2020). Interestingly, the MAT and MAP for several towers exceeded the global distribution (Fig. 2) which could be due to errors in reporting, or incomplete synthesis of global MAT and MAP distributions in WorldClim 2.1 as discussed in more detail below. The peak of the tower MAT distribution was far lower than global MAT sampled from terrestrial regions of the WorldClim2.1 database (Figs. 3 and 4a), but global terrestrial MAT from the WorldClim2.1 sample (8.9 ± 18.5 ℃) was lower than tower MAT 10.5 ± 8.3 ℃ (Table 3) in part because very cold regions were undersampled. Global terrestrial MAP from the WorldClim2.1 sample is drier (739 mm) than towers (averaging 888 mm) (Figs. 3 and 4b; Table 3). Average SRAD across all eddy covariance towers is less than the global mean from terrestrial ecosystems sampled from WorldClim2.1 by over 10% (15241 vs. 13630 kJ m^− 2^ day^− 1^, Figs. 3 and 4c Table 3). Towers also sample areas with lower elevation (averaging 433 m) than the global mean sampled from *elevatr* (645 m) (Fig. 4d; Table 3). All differences between towers and the global distribution of climate and elevation are statistically significant at 𝛼 < 0.05 after applying the Bonferroni correction.

**Table 3:** The mean (± standard deviation) value of global terrestrial climatic and edaphic variables versus those from eddy covariance towers. The global distribution of climatic variables was taken by drawing 10,000 random samples on the sphere from the WorldClim 2.1 database (Fick and Hijmans 2017). The global distribution of soil texture was taken by drawing 10,000 random samples on the sphere from the SoilGrids 2.0 database (Poggio et al. 2021). All differences are statistically significant at 𝛼 < 0.05 after the Bonferroni correction, in bold, except bulk density

Variable	Global	Towers
Elevation (m)	**645 ± 839**	**433 ± 597**
Temperature (℃)	**8.9 ± 18.5**	**10.5 ± 8.3**
Precipitation (mm)	**739 ± 737**	**888 ± 616**
Solar radiation (kJ m^− 2^ day^− 1^)	**15,241 ± 4076**	**13,630 ± 3430**
Sand (%)	**44.7 ± 14.0**	**41.5 ± 18.4**
Silt (%)	**30.5 ± 10.8**	**36.0 ± 12.8**
Clay (%)	**24.8 ± 7.9**	**22.5 ± 9.7**
Bulk density (g cm^− 3^)	1.2 ± 0.003	1.1 ± 0.003
Cation exchange capacity (cmol(c) kg^− 1^)	**25.2 ± 12.3**	**28.5 ± 11.8**
Soil nitrogen (g kg^− 1^)	**0.38 ± 0.32**	**0.58 ± 0.35**
pH	**6.4 ± 1.2**	**6.0 ± 0.9**
Soil organic carbon content (g kg^− 1^)	**5.4 ± 5.9**	**8.3 ± 7.3**
Soil and sedimentary deposit thickness (m)	**14.8 ± 20.0**	**24.0 ± 21.6**

## Soils and uncertainty

**Fig. 5:**
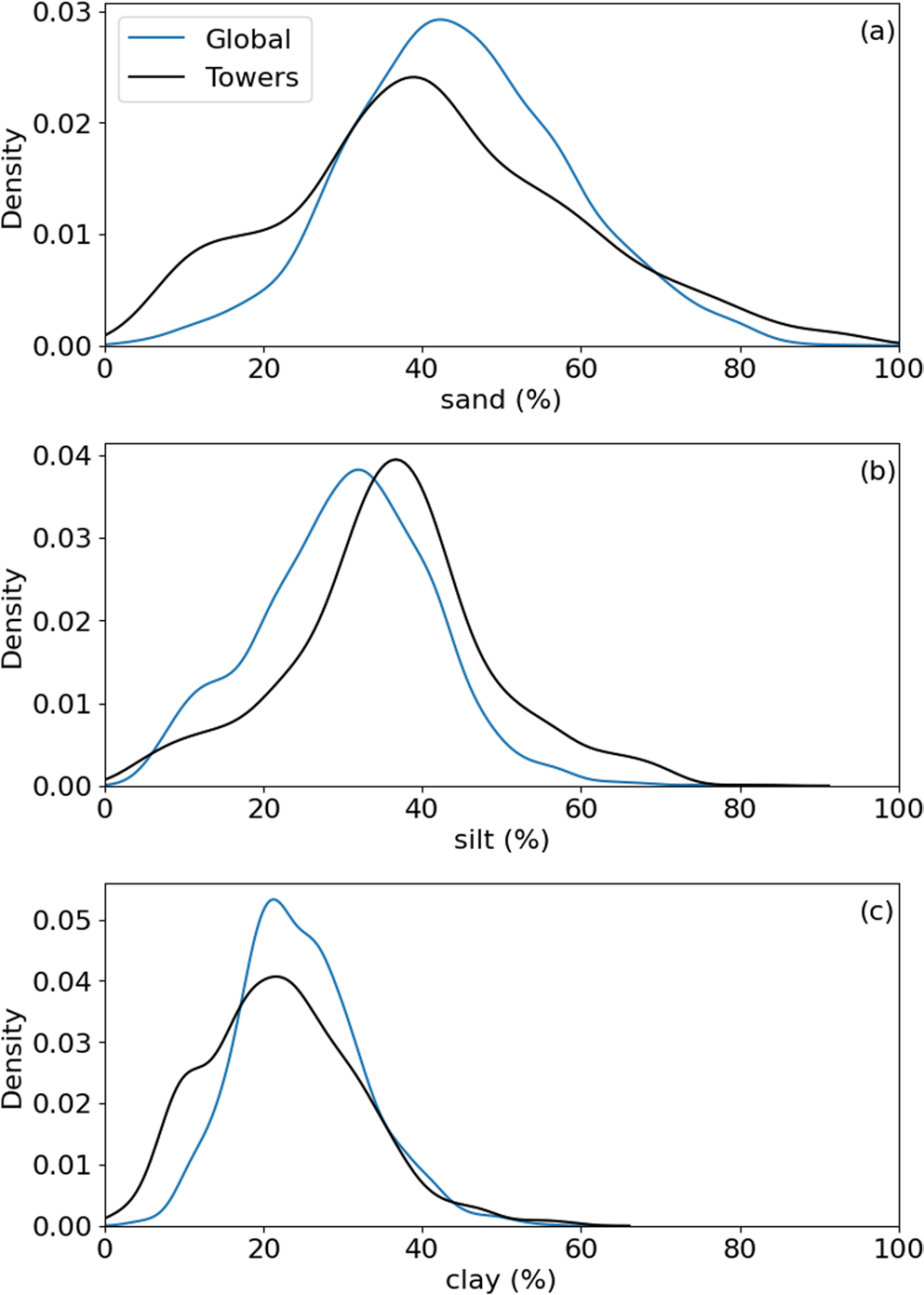
Probability density functions of sand (**a**), silt (**b**), and clay (**c**) fractions for a random sample of 10,000 points on the terrestrial surface from SoilGrids 2.0 (Poggio et al. 2021) (‘Global’) and eddy covariance tower locations (‘Towers’)

Global soils have about 8% more sand and nearly 20% less silt, on average, than the tower database, and these differences are statistically significant (Table 3). Differences in soil texture are reflected in the soil texture triangles (Fig. 4), and the distributions of each soil texture component (Fig. 5) further reveal that the tower networks have a higher mode in average silt content, which points to the suggestion that global towers may sample more fertile soils than the global distribution.

**Fig. 6:**
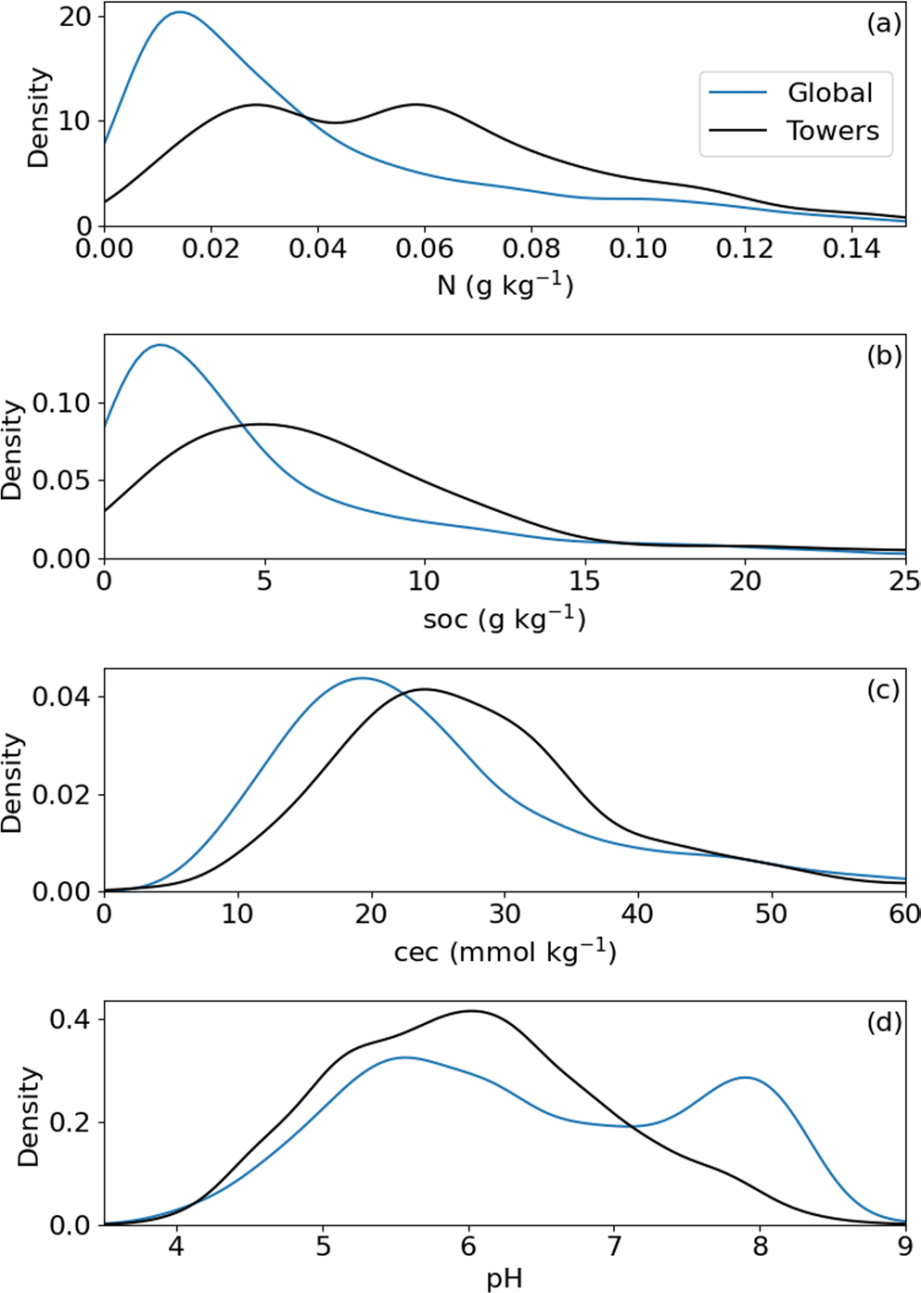
Probability density functions of soil N (**a**), soil organic carbon (soc, **b**), cation exchange capacity (cec, **c**), and pH (**d**) for a random sample of 10,000 points on the terrestrial surface from SoilGrids2.0 (Poggio et al. 2021) (‘Global’) and eddy covariance tower locations (‘Towers’)

**Fig. 7:**
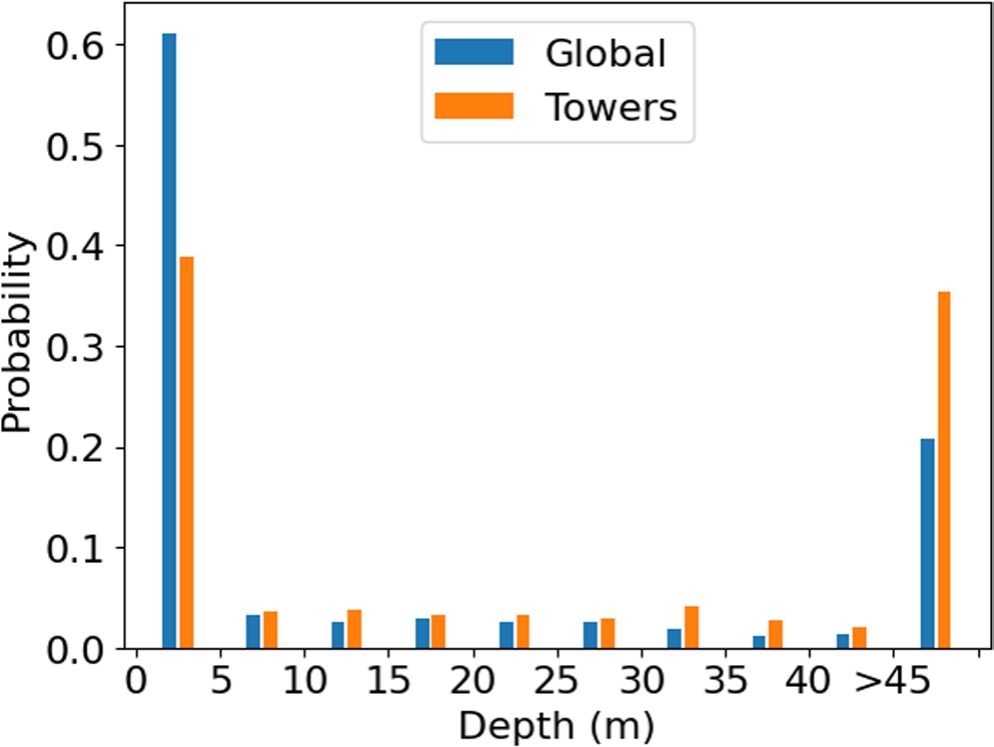
The probability of soil and sedimentary deposit thickness for a random sample of 10,000 pixels, and pixels containing eddy covariance towers, from the database of Pelletier et al. (2016)

Using observations from SoilGrids 2.0, soil nitrogen (N) in the upper 5 cm is ~ 20–25% greater on average across the tower network (0.58 g/kg) compared to the terrestrial land surface (0.38 g/kg) (Table 3; Fig. 7a). Soil organic carbon (SOC) is also greater in the eddy covariance network (8.3 g/kg) than terrestrial soils on average (5.4 g/kg), and average cation exchange capacity is over 10% greater in tower locations than terrestrial soils on average (Table 3; Fig. 7b&c). Average soil pH is slightly more acidic across eddy covariance sites (mean pH ~ 6) than the average value for the terrestrial surface (pH = 6.4) due in part to an undersampling of basic soils with a secondary peak in pH around 8 (Table 3; Fig. 7d). Globally, soil and sedimentary deposit thicknesses of less than 5 m are present in more than 60% of terrestrial ecosystems but less than 40% of the tower network (Fig. 6); mean soil and sedimentary deposit thickness across the terrestrial surface estimated by sampling the database of Pelletier et al. (2016) is 14.8 m but 24.0 m at tower locations (Table 3). Estimates from soils databases suggest that the eddy covariance network samples soils that are deeper and more fertile than the entire terrestrial surface, on average. Differences between climatic and edaphic characteristics for the global versus tower datasets are significant at the 𝛼 < 0.05 level after adjusting for the Bonferroni correction for all variables except bulk density (Table 3).

Soil characteristics can vary considerably across space and across the different datasets used to infer them. For example, across the 14 eddy covariance towers in the CHEESEHEAD19 domain from which soil texture data from cores, the USGS/NRCS database, and the SoilGrids2.0 database were all available (Table 4), SoilGrids2.0 had lower sand content (60.7% versus ~ 75% for the other datasets), higher clay content (9.6% versus ~ 6%), and higher silt content (29.8% versus ~ 19–20%). This is not to say that the soil core and USGS data matched across each individual site: these differed by up to 25% for the case of sand at the SE2 site (Table 4). In brief, one can expect uncertainties when estimating difficult-to-measure edaphic variables that often exhibit considerable heterogeneity (Mulla and McBratney 2002); note for example that the USGS/NRCS database estimates sand content that ranges from 92.7% at NE2 to 49.5% at SE2, a difference of over 40% within the 10 × 10 km CHEESEHEAD19 domain (Table 4).

**Table 4:** Soil texture characteristics from the CHEESEHEAD19 experiment (Butterworth et al. 2021) inferred from soil cores within the eddy covariance flux footprint, the USGS/NRCS database at the flux tower locations (Shveytser et al., 2024), and the SoilGrids 2.0 database (Poggio et al. 2021) at the flux tower locations

Site Name	Cores			USGS			SoilGrids		
	sand	silt	clay	sand	silt	clay	sand	silt	clay
NW1	83.0	11.8	5.2	68.4	21.1	10.5	57	32.8	10.2
NW2	77.3	14.3	8.4	65.1	29.5	5.4	57.7	31.7	10.6
NW3	NA	NA	NA	56.4	32.7	10.9	56.7	32.2	11.1
NW4	NA	NA	NA	56.4	32.7	10.9	56.1	33.2	10.7
NE1	77.2	16.5	6.3	90.2	6.6	3.2	60	29.2	10.8
NE2	79.9	15.1	5.0	92.7	3.6	3.7	62.1	29.3	8.6
NE3	77.3	15.8	6.9	65.1	29.5	5.4	59.1	32.4	8.5
NE4	72.3	20.1	7.6	65.1	29.5	5.4	61.9	29.8	8.2
SW1	71.6	21.2	7.2	65.1	29.5	5.4	59.7	28.5	11.9
SW2	77.1	19.2	3.6	90.2	6.6	3.2	56.9	31.1	12
SW3	84.2	13.6	2.2	65.1	29.5	5.4	57.9	32	10.1
SW4	66.7	28.2	5.1	65.1	29.5	5.4	59.3	31.9	8.8
SE2	77.0	19.8	3.1	49.5	42.2	8.3	63.9	27.3	8.8
SE3	67.1	25.5	7.4	79.1	12.4	8.5	63.4	28.2	8.4
SE4	NA	NA	NA	56.4	32.7	10.9	62.9	28.6	8.6
SE5	71.1	21.1	7.8	90.2	6.6	3.2	66.6	25.4	7.9
SE6	71.3	22.3	6.4	90.2	6.6	3.2	63.8	27.2	9

Further to the point of data uncertainty, the mean (± standard deviation) difference between tower-reported and WorldClim 2.1 MAT values is 0.27 ± 2.29 ℃ with a maximum difference, somehow, of 32 ℃ (Table S1). The mean difference in MAP between tower-reported and WorldClim 2.1 information is 28.3 ± 361 mm with a maximum difference of, remarkably, 8678 mm (Table S1). These differences arise for multiple reasons including clerical errors when creating eddy covariance metadata, uncertainties in reporting, uncertainties in tower measurements, uncertainties in climate datasets, and differences in the time period covered by climate normals and eddy covariance tower measurements or reports.

## Discussion

### Tower Location

There are key geographic and edaphic features of the eddy covariance networks (Xiao et al. 2012; Chu et al. 2017; Pastorello et al. 2020), which tend to sample ecosystems in Europe, Australia, and North America (Table 2) with fewer sites in tropical, dry, and cold climates (Figs. 1 and 2 & 2) and less fertile and deep soils (Table 3; Figs. 4, 5, 7 and 6). More eddy covariance research opportunities to study tower environments exist now that the NEON, AmeriFlux, ICOS, TERN and other measurement networks provide baseline eddy covariance data from relatively well-studied ecosystems (Hargrove et al. 2003; Schimel et al. 2007; Rebmann et al. 2018; Cleverly et al. 2019; Heiskanen et al. 2022). As currently operating, these networks will not resolve global discrepancies in the geographic distribution of flux towers (e.g. Figure 1) as they mostly add observational capacity to the United States, Europe, and Australia (Table 2), but all towers provide critical information for our understanding of surface-atmosphere exchange (Baldocchi 2008; Chu et al. 2017). There is a growing need to understand trace gas, water, and energy fluxes across global ecosystems that will be essential for understanding nature-based climate solutions and quantifying baselines, additionality, and permanence metrics required of carbon credit markets (Hemes et al. 2021; Novick et al. 2022; Seddon 2022). The tendency of towers to be close to a neighboring tower (Figure S1) can help understand how different ecosystems behave when subject to similar climatic conditions in a ‘paired’ tower design (Baldocchi 2014), but geographic discrepancies also point to a lack of representativeness for understanding fluxes across large regions (Hargrove et al. 2003; Schimel et al. 2007).

### Uncertainty

Climate changes dynamically across time and space. The use of MAT and MAP in our analysis therefore introduces a shifting baseline as climate continues to change, but we know of no way to encapsulate the distribution of climatic variables that is more commonly used or straightforward. These variables may be prone to reporting or clerical uncertainty as suggested by Fig. 2 and the comparison of MAT and MAP values in the *Results* where it was noted that MAT (MAP) differences between tower reporting and global databases differ by up to 32 ℃ (8678 mm). From this analysis it should be clear that global climate datasets reasonably simulate tower climatic conditions in many instances, but both tower and climate dataset information should be double-checked for logic when reporting; we use reported values here to help quantify the degree of uncertainty that climate reporting and extrapolation can introduce. Uncertainties in climate data reporting are unlikely to impact our overarching results as it is clear from the global distribution that fewer towers exist in regions both hot and cold, and wet and dry (Figs. 2 and 3) as characterized in previous studies (Pastorello et al. 2020). Effective sampling is critical for upscaling sparse tower networks to estimate fluxes at continental scales while minimizing uncertainty (Papale et al. 2015).

### Soils

The challenge of simplifying edaphic characteristics is less straightforward as these vary – often dramatically – across space and soil depth, and sometimes across time in the case of extreme events or management, all of which impact how we interpret eddy covariance observations (Oren et al. 2006; Vanderborght et al. 2010; Tuovinen et al. 2019; Levy et al. 2020; Herbst et al. 2021; Rey-Sanchez et al. 2022). Data products like SoilGrids 2.0 represent the state-of-the-art for understanding of the global distribution of soil characteristics, but from our analysis substantial uncertainties result when applying such data to individual tower locations (Table 4), noting that we only studied the top 5 cm of soil structure and selected soil chemical attributes for simplicity. Whereas such uncertainties inevitably impact our complication of edaphic characteristics at each tower (Figs. 4, 5 and 7; Table 4) and therefore need to be considered when interpreting results, we feel that the overarching result that eddy covariance towers sample regions of the globe with richer soils is robust. The lack of measurement towers across deserts and other dryland regions (e.g. Figure 1) and a proportionate number of towers in croplands (which, admittedly, are disproportionately important for human health and wellbeing, Table 1) suggest that the full distribution of soil fertility is poorly captured. Global analyses and upscaled data products should be cognizant of this edaphic bias. We note that there is a large undersampling of ecosystems with acidic soils, especially those with pH ~ 8 that are buffered by calcium carbonate, leaving opportunities to study the impacts of abrupt spatial heterogeneity in soil characteristics including pH that are largely controlled by aridity (Slessarev et al. 2016).

### Metadata and information availability

It is apparent from Table S1 that key information is unavailable for some sites, which stands to reason if tower data and metadata are often shared on a volunteer basis (Novick et al. 2018) that may have no mandate or additional funding to support data sharing. We advocate for additional information sharing for datasets for which sharing is allowed and look forward to new FLUXNET synthetic activities that build off the legacy of the Marconi (Falge et al. 2006), La Thuile (Verma et al. 2014), and FLUXNET2015 (Pastorello et al. 2020) global datasets.

## Conclusions

Eddy covariance studies arise to address pressing questions in surface-atmosphere exchange. Only recently have towers been sited at locations with large-scale representativeness in mind (Schimel et al. 2007), so one should not expect that towers across the globe have representative distributions across climatic and edaphic space. The current tower bias toward temperate regions and more fertile soils leaves opportunities to expand the flux network globally and further build international partnerships to help do so; at a minimum we hope that our results lead to additional consideration of edaphic conditions and site-level reporting of soil characteristics. Opportunities to study a richer suite of global edaphic variability across the flux network certainly exist, and we hope that our synthesis helps the community understand the importance of soil when inferring regional or global patterns from eddy covariance datasets.

## Electronic Supplementary Material

Below is the link to the electronic supplementary material.

Supplementary Material 1

## Data Availability

Code for data analyses in the present manuscript is available athttps://colab.research.google.com/drive/1OHs0T5vT1n8mIkHxP_7CPTTL7izL6HyBand https://zenodo.org/records/18894391
