# Supplementary material for: The spatial and environmental distribution of the global eddy covariance tower network
Source: Int J Biometeorol. 2026 Mar 30;70(4):108. doi: 10.1007/s00484-026-03183-8 (PMC13035709; doi:10.1007/s00484-026-03183-8)
Supplement: Supplementary file 1 [file 484_2026_3183_MOESM1_ESM.docx]

Supporting Information

Figure S1. The distance between each eddy covariance tower in the database described in Table S1 to its nearest tower neighbor, plotted as the sorted cumulative sum of all sites.

Table S1. A database of eddy covariance tower locations and associated environmental variables. Climate data from databases was obtained from WorldClim 2.1 (Fick and Hijmans, 2017). Soils data were obtained from the top 0-5 cm depths from the SoilGrid 2.0 database (Poggio et al., 2021). MAT = mean annual temperature. MAP = mean annual precipitation. SRAD = solar radiation. bdod = bulk density. cec = cation exchange capacity. N = soil nitrogen content. pH = soil pH. soc = soil organic carbon content. 

See [https://github.com/paulstoy/FluxTowers/blob/main/Towers.csv](https://github.com/paulstoy/FluxTowers/blob/main/Towers.csv) 

and [https://zenodo.org/records/18894391](https://zenodo.org/records/18894391)

Table S2. Soil and sedimentary deposit thickness of pixels that contain eddy covariance towers following Pelletier et al. (2016).  

See [https://github.com/paulstoy/FluxTowers/blob/main/TowerSoilDepth.csv](https://github.com/paulstoy/FluxTowers/blob/main/TowerSoilDepth.csv) 

and [https://zenodo.org/records/18894391](https://zenodo.org/records/18894391)
